# Comparative Clinical Study on Magnesium Absorption and Side Effects After Oral Intake of Microencapsulated Magnesium (MAGSHAPE^TM^ Microcapsules) Versus Other Magnesium Sources

**DOI:** 10.3390/nu16244367

**Published:** 2024-12-18

**Authors:** David Pajuelo, Justyna M. Meissner, Teresa Negra, Alan Connolly, Jose L. Mullor

**Affiliations:** 1 Bionos Biotech SL, LabAnalysis Life Science, Biopolo Hospital La Fe, 46026 Valencia, Spain; jmeissner@bionos.es (J.M.M.); jlmullor@bionos.es (J.L.M.); 2 Lubrizol Life Science, 08850 Gavà, Spain; teresa.negra@lubrizol.com (T.N.); alan.connolly@lubrizol.com (A.C.)

**Keywords:** magnesium, bioavailability, absorption, plasma, sustained release, food supplement, side effects

## Abstract

Background/Objectives: Magnesium (Mg)-based food supplements contribute to the maintenance of adequate levels of Mg that are essential for overall health and well-being. The aim of this double-blind, randomized, cross-over clinical study was to assess the plasma Mg levels in volunteers following the oral administration of a magnesium-based nutraceutical ingredient, MAGSHAPE^TM^ microcapsules (Mg-MS), in comparison to other commonly used magnesium sources, including the following: Mg Oxide (MgO), Mg Citrate (Mg-C), and Mg bisglycinate (Mg-BG). Methods: A total of 40 healthy women and men were put on a low-Mg diet for 7 days, and after 8 h of fasting, a blood sample was taken from a digital puncture before (0 h) and 1 h, 4 h, and 6 h after the oral intake of each product. Results: Our results showed that the blood plasma levels of Mg increased significantly at all tested time-points after the oral intake of Mg-MS, while the blood plasma levels of Mg increased significantly only after 1 and 4 h of the oral intake of MgO and Mg-C, respectively. However, no significant increase in Mg levels was observed upon the intake of Mg-BG. Interestingly, the Mg-MS microencapsulation technology was observed to enable a sustained increase in plasma Mg levels over the duration of this study, i.e., 1, 4, and 6 h after oral intake. A direct comparison of the increase in plasma Mg levels over the 6 h period revealed that the Mg-MS microencapsulation technology significantly increased Mg bioavailability compared to the non-microencapsulated MgO. Our study also showed that, compared to the other Mg sources tested, the Mg-MS microencapsulation technology reduced adverse side effects commonly associated with Mg supplementation, specifically with regard to increased intestinal motility and sensations of gastric heaviness following oral administration. Conclusions: Altogether, this clinical study introduced MAGSHAPE^TM^ microcapsules as a bioavailable and well-tolerated alternative to existing Mg-based ingredients used in food supplements.

## 1. Introduction

Magnesium (Mg) is a vital mineral and an essential element in the biochemistry of living organisms. It plays a crucial role as a cofactor in over 600 enzymatic reactions, including those involved in energy production, DNA synthesis, and protein synthesis [1]. A normal adult has approximately 1000 mmol of corporal Mg (22–26 g), and 99% of this Mg is stored within intracellular compartments. Of that total, 60% is stored in bones, 20% in muscles, and another 20% in other tissues. Just 1% of total corporal Mg is in the extracellular milieu and present as bioavailable Mg. Of this 1%, 60% is in a free/ionized form, 10% is ligated, forming complexes to different salts (citrate, phosphates, oxalate, and other anions), and the remaining 30% is bound to proteins [2,3]. The presence of free Mg in blood is the key regulator of Mg excretion via kidney [4]; in particular, 80% of Mg in plasma is filtered by the glomerulus, of which 95% is reabsorbed by the nephron [2,3]. Of the total dietary Mg consumed, only about 24–76% is mainly absorbed in the ileum and colon, and the rest is eliminated in the feces [4].

In humans, Mg is particularly important for maintaining normal muscle and nerve function, regulating blood glucose levels, inflammation, response to cancer, and infectious diseases and supporting a healthy immune system [5,6]. It also contributes to the structural development of bones and is required for the synthesis of the antioxidant glutathione [5,6]. A deficiency in Mg may lead to mild to severe physiological problems, such as muscle cramps, fatigue, cardiac arrhythmias, biochemical abnormalities of hypokalemia and hypocalcemia, hypertension, atherosclerotic vascular disease, altered glucose homeostasis, and metabolic bone disease [7,8]. Therefore, maintaining adequate levels of Mg is critical for overall health and well-being. Supplementation with Mg is common to prevent or treat Mg deficiency and to support various bodily functions. Mg is available in several forms, including Mg oxide (MgO), Mg citrate (Mg-C), Mg bisglycinate (Mg-BG), and Mg chloride, each having different absorption rates and potential effects on the body [9,10,11,12]. Mg supplements are often recommended for individuals who have difficulty consuming enough Mg through their diet, such as those with gastrointestinal disorders, certain chronic conditions, or increased physiological needs like pregnancy or intense physical activity.

In both the food and pharmaceutical industries, there is a growing interest in the development of microencapsulation techniques to effectively deliver various bioactive ingredients, enabling the development of innovative food supplements with improved functional properties and enhanced nutritional value [13]. The microencapsulation of actives which are applied in food supplements can prevent their degradation or modification, enhance their solubilization, and control and regulate their delivery after consumption [13,14,15].

Several approaches can be used to evaluate Mg bioavailability upon the oral intake of food supplements. Urinary Mg levels provide an understanding of overall Mg metabolism, reflecting the body’s regulatory mechanisms and excretion rates [16]. On the other hand, plasma Mg levels are directly associated with intestinal absorption efficiency, making them particularly relevant for investigating the early phase of Mg uptake, such as in the present work, where we investigated the effects of microencapsulation technology on absorption dynamics. Therefore, we performed a detailed comparative analysis of the Mg levels in plasma upon the oral intake of different Mg ingredients used for food supplementation and MAGSHAPE^TM^ microencapsules (Mg-MS). Our study was complemented with a comprehensive subjective self-assessment questionnaire about the tested samples by the volunteers. The results of the present clinical study showed that Mg-MS significantly enhances Mg levels relative to other commonly used Mg supplements and is well-tolerated by the volunteers.

## 2. Material and Methods

### 2.1. Tested Products

The efficacy of the tested samples was evaluated in a clinical study conducted at the facilities of Bionos Biotech S.L. The study protocol was approved by the Ethical Committee for Medical Research from Hospital La Fe, Valencia, Spain (date: 20 December 2023; code number: 2023-1181-1), and the clinical study was registered in the database of ClinicalTrials.gov (ID: NCT06225349). The samples under investigation were four food supplements composed of the same ingredients but with different elemental Mg sources: Mg oxide (MgO), Mg citrate (Mg-C), Mg bisglycinate (Mg-BG), and MAGSHAPE^TM^ microcapsules (Mg-MS), a patent pending ingredients based on the micronization and microencapsulation of MgO in a food-grade matrix to enhance its absorption and improve its rheological properties. MAGSHAPE^TM^ is a trademark of The Lubrizol Advanced Materials, Inc. srl (Gavà, Spain) or its affiliates. To properly assess the different sources of Mg, each Mg source was prepared as an orosoluble powder for consumption by the panelists. This format is increasingly popular among consumers seeking alternatives to the more traditional tablets and capsules. This format also allows the solubilization and subsequent ionization of the Mg upon ingestion by the acid environment of the stomach. Each sample was supplied in an opaque glass bottle with individual doses containing 375 mg of elemental Mg. The powder was placed directly in the mouth by each volunteer and ingested without water. After ingestion, volunteers were provided with a small amount of water to aid in swallowing and ensure complete consumption.

### 2.2. Study Population

The selected panelists represent the target healthy population with normal plasma Mg levels seeking to maintain adequate Mg levels to support daily physiological processes such as relaxation, improved sleep, sports nutrition, and post-workout recovery. While studying populations with Mg deficiencies or increased demands could provide additional insights, our goal was to assess the efficacy of the different Mg sources in maintaining healthy Mg plasma levels and highlight their ability to prevent Mg deficiency in the target population.

A total of 40 healthy volunteers between 20 and 55 years old, men and women of all races/ethnicities, with a body mass index of 18–35 kg/m^2^, were enrolled in this clinical study. The exclusion criteria for the selection of volunteers were as follows: (i) individuals with gastroendocrine and gastrointestinal diseases (diabetes insipidus, gastritis, Crohn’s disease, celiac disease, ulcers, intolerances, etc.); (ii) individuals with cardiorespiratory diseases (chronic bronchitis, chronic obstructive pulmonary disease, pulmonary emphysema, asthma and bronchiectasis, thrombi, heart disease, heart disorders, arrhythmias, insufficiencies, etc.); (iii) pregnant or lactating women or who plan to become pregnant during the study; (iv) individuals under medical treatment in the weeks prior to the study that could interfere with the evaluations of the present study (according to the investigator’s criteria); (v) individuals who were within a dietary period outside their usual habit; (vi) individuals who were within a dietary period where cannot follow the diet restrictions needed to run this study; (vii) individuals who demonstrated manifest incapacity to understand or follow the protocol or the informed consent; (viii) individuals with allergy or reactivity to any of the components; (ix) individuals surgically operated for a heart condition; (x) individuals forecasted to have a changing routine or relevant way of life, during the period of this study; (xi) individuals that participated in a clinical study of this type, at least 15 days before the start of this study. Additionally, volunteers were asked to avoid the consumption of food supplements, nutricosmetics, or specific food containing high levels of Mg (Table S1) during the week before the start of each treatment and during the study. One of the volunteers did not complete the study due to adverse effects upon the oral intake of Mg-BG, and two volunteers did not complete the study due to personal reasons; all these three volunteers were replaced, and the data of the replaced volunteers were not considered for the analysis. The information about the volunteers is detailed in Table S2.

### 2.3. Treatment

This study was a randomized, double-blind trial that aimed to compare the increase in Mg in plasma after the oral intake of different Mg sources. This study was divided into 4 stages based on single-day sampling at 7-day intervals. Before starting and between stages, the volunteers followed a low-Mg diet for 1 week, excluding Mg-rich foods as described in Table S1 and drinking only low-mineralized water. After 8 h of fasting, blood samples were obtained by finger prick (0 h) and 1 h, 4 h, and 6 h after a single oral intake of the corresponding Mg product. This 1-week procedure was repeated for all 4 tested Mg products.

For the assignment of the Mg sources in each stage, volunteers were randomly divided into four groups. Randomization was conducted using a computer-generated random number table. Every 7 days the volunteers received one of the samples based on this randomized order. This was repeated until each volunteer had tested all the samples. All the tested products were labeled as Product A, Product B, Product C, and Product D; neither the responsible technical personnel nor the volunteers had access to the real names of the products.

During each sampling day, volunteers followed a standardized low-Mg diet, consisting of a ham and cheese sandwich after the 1 h time point, and low-mineralization water ad libitum. This study did not pose any risks for the volunteers, since all Mg forms used in this study are widely used at the food level in numerous pharmaceutical preparations and dietary supplements, and elemental Mg was administered in a single dose of 375 mg, according to Regulation (UE) No 1169/2011 (100% of NVR, EFSA).

### 2.4. Sample Preparation

Blood samples were taken by digital puncture (non-invasive fingerstick procedure, by using VeriFine safety lancets 21G) for each of the 40 volunteers. Blood samples were collected in EDTA-containing tubes (Minicollect tube K3E EDTA), centrifuged at 1000 rpm, 5 min at 4 °C, and plasma (supernatant) was collected and stored at −20 °C.

### 2.5. Magnesium Quantification in Plasma

The amount of Mg in the samples was determined with an inductively coupled plasma quadrupole mass spectrometer Agilent 7900 Series ICP-MS (Agilent, St. Clara, CA, USA), equipped with platinum cones, a Micromist concentric nebulizer, a Scott-type spray chamber, an off-axis double lens system, a hyperbolic quadrupole as a mass filter, and a collision/octopolar reaction cell operating in collision mode using Helium gas. ICP-MS was successfully implemented in the past for Mg quantification [17,18,19,20,21].

The reagents used to prepare calibration line in the 0–2000 μg/L range were ICP Mg standard of 10 mg/mL (High Purity Standard, Charleston, SC, USA) and ICP scandium standard solution at 20 μg/g (ISC Science, Paris, France), employed as internal standard. Mass calibration and resolution adjustment were performed daily by using the AutoTune function of ICP-MS MassHunter software version 5.2, For ICP-MS, a daily performance check was performed by using a solution containing 1 μg/L Li, Mg, Co, Y, Tl, and Ce in 2% (*v*/*v*) HNO_3_. The formation of oxides (^156^CeO^+^/^140^Ce^+^ < 1.5%) and double charges (^140^Ce^2+^/^140^Ce^+^ < 3%) were minimized by adjusting the nebulizer gas flow, and the autolens voltage was adjusted by the transmission of ^7^Li, ^89^Y, and ^205^Tl ions. According to the manufacturer’s operating conditions in typical work conditions, the sensitivities of ^7^Li, ^89^Y, and ^205^Tl were higher than 3000, 10,000, and 6000 cps, respectively, with an RSD value of less than 5%.

The monitored isotopes were ^24^Mg and ^45^Sc, and the instrumental acquisition parameters to Mg determination were as follows: 15 L/min plasma gas flow rate; 1 L/min carrier gas; 1550 W radio frequency power and 1.80 V of RF matching; 0.3 rps nebulizer pump speed. A total of 10–50 µL of the sample were taken and digested with nitric acid (69%, *w*/*w*) for 24 h at room temperature, obtaining a final volume of 5 mL with ultrapure water. The solutions were filtered with a 0.45 µm pore size nylon filter.

To selectively reduce or remove polyatomic interferences, the dynamic reaction cell (DRC) was used in collision mode with Helium, a non-reactive gas, in combination with kinetic energy discrimination (KED). At equal mass, polyatomic ions are larger and collide with the cell gas more frequently than analyte ions. In collisions, polyatomic ions either break up, eliminating interference, or lose energy and are removed from the ion beam. The obtained correlation factor R of the calibration curve was equal to or greater than 0.9999. After the analysis of the samples, one of the standards was analyzed again as an unknown sample so that the relative standard deviation (RSD) between the theoretical and calculated concentration was less than 5%. The results were expressed as mg L^−1^.

### 2.6. Self-Assessment Questionnaire

Different parameters associated with the products were subjectively evaluated by the volunteers with self-assessment questionnaires (Supplementary Materials). In this self-assessment questionnaire, different parameters were evaluated by a subjective category, which is indicated for each specific question. These questionnaires were completed by all volunteers for all the products, gathering information right after the oral intake and at the end of the day.

### 2.7. Statistical Analysis

Data were statistically analyzed by one-way ANOVA test and Dunnet’s post hoc multiple comparisons test. Data outliers were identified considering the entire data set and excluded by using a ROUT of 5. Statistical significance was declared at *p* < 0.05, 95% of confidence. Bars in the charts represented the mean value for each condition, and error bars indicated the standard error of the mean (SEM) for each group of values. The sample size of 40 volunteers was determined based on a pilot study conducted prior to this clinical trial. The pilot study assessed magnesium levels in plasma after the oral intake of the same tested samples, following identical experimental and analytical conditions. Statistical analysis of the pilot study results indicated that 40 participants were sufficient to detect significant differences, ensuring adequate statistical power for this study. Baseline values from all volunteers and samples were merged together and used as common baseline value pool for all samples. Pooling baseline values is a common approach reported in similar studies [22,23,24,25,26], proven to reduce statistical noise by creating a larger, more robust dataset and minimizing the impact of small, random fluctuations in individual baseline. This method ensures consistency and comparability across treatments and accounts for natural week-to-week variations in magnesium levels caused by uncontrollable factors. The area under the curve was calculated using the trapezoidal method, which estimates the value of the area under the curve by summing the areas of trapezoids formed between successive data points [26,27,28]. All statistical analyses, including the calculation of the area under the curve, were performed with GraphPad Prism v4.

## 3. Results

### 3.1. Mg Levels in Blood Plasma upon Oral Intake

**Figure 1:**
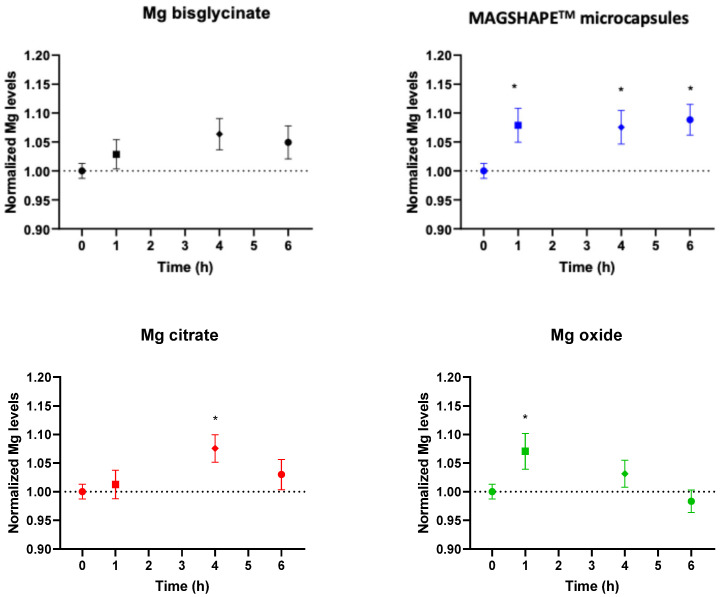
Graphical representation of the Mg levels in plasma (normalized to basal time; 0 h) in all volunteers in the assessed time points for each sample. Dotted line represents the normalized basal levels to time 0 h. Data are represented as mean ± S.E.M. Asterisks indicate statistically significant differences compared to basal levels (0 h) as * *p*-value < 0.05.

We evaluated the Mg levels in blood plasma upon the oral intake of a microencapsulated MgO ingredient (Mg-MS), compared with other common Mg forms such as Mg oxide (MgO), Mg citrate (Mg-C), and Mg bisglycinate (Mg-BC). Figure 1 shows the Mg levels in plasma for each sample at each time point, normalized to the baseline (Time 0 h, before oral intake) levels. Data expressed as mg L^−1^ are shown in Figure S1. When volunteers ingested Mg-MS, the levels of Mg in plasma increased at all tested time-points; specifically, Mg levels increased by 7.9%, 7.5%, and 8.8% after 1 h, 4 h, and 6 h of the oral intake, respectively (Figure 1 and Figure S1). On the other hand, the increase in plasma Mg after oral intake of Mg-BG was not significant at any of the time points evaluated. Oral supplementation with Mg-C increased Mg levels in plasma after 4 h by 7.5%, while Mg levels increased at 1 h by 7.1% upon oral intake of MgO (Figure 1 and Figure S1).

The data of the quantification of Mg levels for each sample were represented as curves, as shown in Figure S2, including both the absolute (mg L^−1^) and normalized data to the basal (time 0 h) level. Mg levels in plasma have different dynamics depending on the tested samples. MgO showed a short-term increase in the Mg levels, corroborated by the *p*-value < 0.05 (Figure 1 and Figure S1), while Mg-C and Mg-BG seemed to increase mean Mg levels at a later time-point; however, these values were not significant, with the exception of Mg-C at 4 h (*p*-value < 0.05, Figure 1 and Figure S1). Interestingly, the dynamics of plasma Mg levels after the oral intake of Mg-MS was different to the others Mg sources tested. In particular, Mg-MS increased Mg levels in the short term (1 h after the ingestion) and maintained plasma Mg levels significantly higher than the basal time at least up to 6 h (Figure 1 and Figure S1).

**Figure 2:**
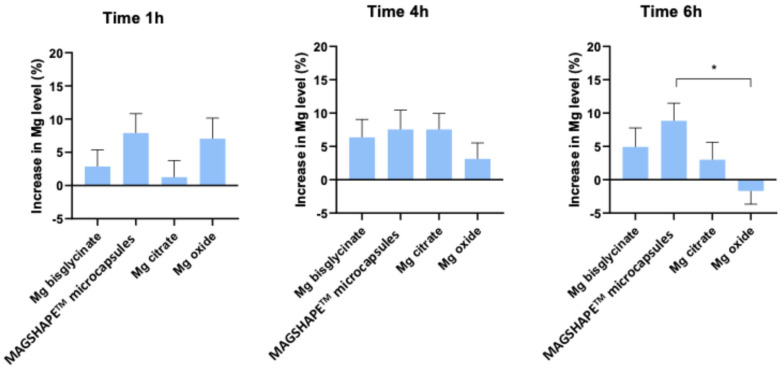
Bar graph representation of the increase in Mg levels in plasma in percentage (%) after 1, 4, and 6 h of the oral intake compared to the basal levels for each tested product. Data are represented as mean ± S.E.M. Asterisks indicate statistically significant differences compared to basal levels (0 h) as * *p*-value < 0.05.

In order to directly compare the efficacy of the different Mg sources, we correlated to what extent each sample increased Mg levels in plasma upon oral intake. For this, we represented the average increase in Mg levels in plasma after 1 h, 4 h, and 6 h of oral intake compared to averaged corresponding basal levels (time 0 h, common baseline) for each Mg source. Then, these average increases were statistically compared between themselves. As observed in Figure 2 and Figure S3, no differences in the increased Mg levels in plasma were found between the tested products after 1 h or 4 h of oral intake (in all pair-wise comparisons). However, when we compared the increase in Mg absorption after 6 h of the oral intake, the increase induced by Mg-MS was 10.3% higher than that observed for MgO (Figure 2 and Figure S3).

**Figure 3:**
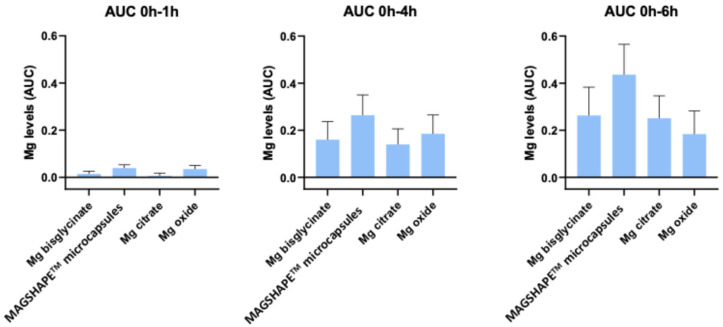
Graphical representation of the area under the curve (AUC) in the whole study period (0 h to 6 h) and in the indicated time intervals for each product.

In order to compare the effect of the tested Mg sources on the plasma Mg levels in more detail, we delimited different time intervals in the curves represented in Figure 3 and calculated the area under the curve (AUC) as a measure of the total amount of Mg in plasma. In particular, we calculated the AUC for the whole study period (i.e., 6 h) and for the following time intervals: 0 h–1 h, 0 h–4 h, and 0 h–6 h. Then, the calculated AUCs were compared between the tested samples (Figure 3). Our results showed that there were no statistically significant differences between the calculated AUCs of the tested Mg sources, independent of the analyzed time interval (Figure 3).

### 3.2. Subjective Evaluation of the Tested Products

**Figure 4:**
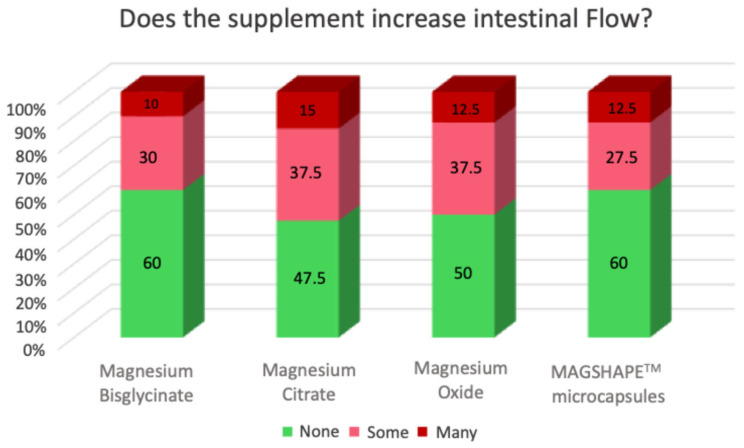

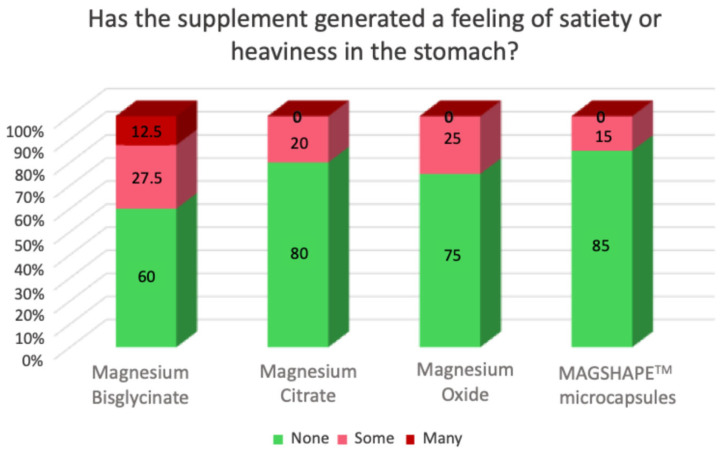
Graphical representation of the results (regarding side effects) of the self-assessment questionnaire at the end of the day after the oral intake of the Mg sources.

**Figure 5:**
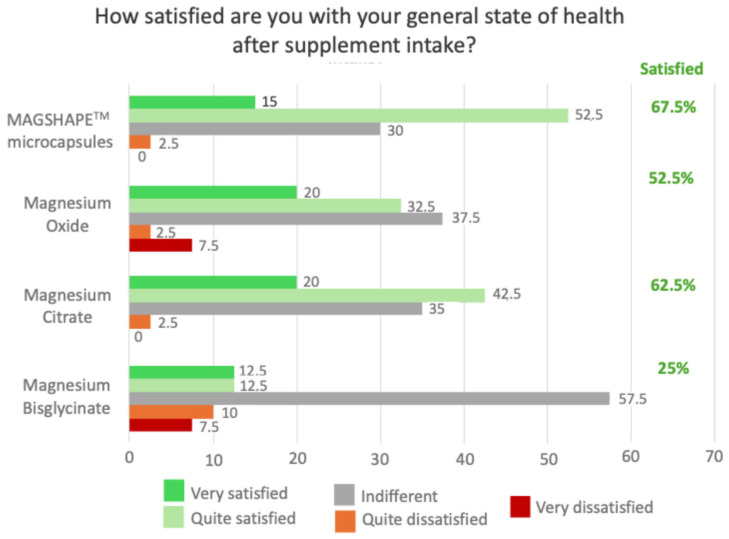
Graphical representation of the results (regarding satisfaction rate) of the self-assessment questionnaire at the end of the day after the oral intake of the Mg sources.

The adverse side effects and perception of the different Mg sources were subjectively evaluated using a test (self-assessment questionnaire) immediately after the oral intake of each sample and at the end of each day to evaluate their potential side effects several hours after oral intake. The results obtained at the end of the day of the oral intake are presented in Figure 4 and Figure 5. Our results showed that, compared to Mg-MS, volunteers reported increased intestinal flow with MgO and Mg-C and increased stomach heaviness with Mg-BG and MgO (Figure 4). Moreover, 67.5% of the volunteers reported that they were satisfied with their state of health after the consumption of Mg-MS, a percentage higher than the other Mg sources (Figure 4 and Figure 5); on the other hand, 70% of the volunteers reported that they were either very satisfied or quite satisfied with the format of the Mg-MS product, a percentage higher than the other Mg sources (Figure 4 and Figure 5).

## 4. Discussion

In this clinical study, we aimed to evaluate and compare the rate of intestinal absorption of different sources of Mg. This was achieved by monitoring Mg levels in the plasma of healthy human volunteers upon the oral intake of a microencapsulated Mg-based ingredient, Mg-MS, compared to other commonly used Mg sources. As already mentioned, the measurement of urine Mg levels provides valuable insights into Mg metabolism, while the measurement of plasma Mg levels best reflects the primary focus of this study, which was to evaluate the intestinal absorption of Mg from the different samples. Currently, there are a variety of Mg supplements available to prevent hypomagnesemia, allowing people to maintain healthy Mg levels and improve their overall health. Among these, MgO, Mg-C, and Mg-BG are the Mg sources most used in Mg-based food supplements with efficacy in increasing the Mg blood plasma levels that were previously demonstrated [9,10,11,12]. Mg-BG is based on the chelation of the Mg with two molecules of glycine, an amino acid that enhances its solubility and absorption in the digestive system [29]. Mg-C is another widely used form of organic Mg known for its high solubility and ability to increase Mg levels, comparable with or even higher than some inorganic forms [30,31]. On the other hand, one of the most commonly used forms of Mg in dietary supplements is MgO. Its main advantage is its high elemental Mg content, which allows manufacturers to deliver significant amounts of Mg in relatively small dosages. However, MgO exhibits low solubility, leading to limited efficacy in some clinical studies [32,33].

The results of the quantification of Mg levels in plasma are in agreement with previous observations that reported an increase in plasma Mg concentration following a single oral administration of Mg [34,35]. As expected, the tested Mg-based samples presented different kinetics in terms of the increase in Mg levels in plasma [12]. The oral intake of Mg-C increased Mg levels in plasma 4 h after the oral intake, in contrast to MgO, which increased Mg levels only after 1 h. These results indicated that the inorganic and organic forms of Mg were absorbed and eventually transported into the bloodstream by following different dynamics, probably due to their differences in their chemical nature. For example, the peak at 4 h observed for Mg-C is most likely the result of a delayed absorption of the organic Mg from the intestinal tract [34] compared to MgO. Depending on the need of a particular individual, they might require a fast-absorption Mg supplement or a supplement that increases Mg levels in a more sustained manner. Based on our results, Mg-C and MgO could be suitable Mg sources depending on the specific needs of the individuals. However, neither Mg-C nor MgO demonstrated an increase in plasma Mg levels 6 h post oral administration. This indicated that neither sample effectively induced sustained elevation in Mg levels, thereby limiting their applicability for interventions aimed at achieving prolonged Mg supplementation. Most importantly, the microencapsulated Mg-MS increased Mg levels in plasma at all assessed time-points, i.e., after 1, 4, and 6 h of the oral intake. This finding highlighted the efficacy of Mg-MS microcapsules, combining both a short-term increase in the Mg levels in plasma together with the sustained increase in Mg levels up to, at least, 6 h.

The observed sustained release dynamics of Mg levels in plasma upon the intake of Mg-MS might have important beneficial implications for the physiology of the organisms. For example, a Mg release over an extended period of time maintains more consistent and stable levels of this mineral in plasma, reducing the frequency of the doses required and extending the beneficial effects of the supplement beyond other Mg-based supplements [34,36]. On the other side, the finding that Mg-MS increases Mg levels as soon as 1 h after oral intake ensures that the Mg-derived beneficial effects are triggered at short-term, in cases where a fast-paced response of the product is expected for a rapid compensation of Mg deficiency [34]. It is worth noting that, in our study, supplementation with Mg-BG did not lead to a significant increase in the Mg levels in plasma at any tested time-point. In this regard, different studies pointed out that a significant increase in Mg levels in plasma is not always achieved after the oral intake of supplements, most likely due to the differences in the pool of volunteers and/or methodologies [34]. The other organic Mg source evaluated in this study, Mg-C, demonstrated an improved efficacy compared to Mg-BG in increasing Mg levels, suggesting that the intestinal absorption of Mg-BG is less efficient. In this regard, the inorganic form MgO demonstrated higher efficacy compared to Mg-BG, as reflected by the short-term increase in plasma magnesium levels observed 1 h post administration, which was not detected upon the oral intake of Mg-BG. Interestingly, our results showed that Mg-MS outperforms Mg-BG in terms of increasing Mg levels throughout the duration of this study.

It is particularly important to examine the comparison between the MgO supplement versus its microencapsulated form, Mg-MS. According to the dynamics over 6 h of the study, both supplements increased the Mg levels at the short term (1 h), but only Mg-MS maintained the Mg levels in plasma significantly higher than the baseline up to, at least, 6 h. This result suggested that the Mg-MS microencapsulation technology applied to MgO not only maintains its rapid absorption and prompt increase in magnesium concentration in plasma but also prolongs its availability in the body for several hours, thereby ensuring a sustained supply of magnesium over an extended period. Indeed, a direct comparison between the increase in Mg levels in plasma from 0 to 6 h revealed that the increase as a result of Mg-MS supplementation is significantly higher than that upon MgO supplementation, demonstrating the efficacy and improvement of the newly developed Mg-MS nutraceutical ingredient.

One of the most common side effects of Mg supplementation are the laxative effects in the form of increased intestinal motility due to the osmotic activity of unabsorbed Mg compounds in the intestine, which stimulates motility [30,31]. Our findings indicated that Mg-MS is associated with a lower incidence of adverse effects compared to the other magnesium sources evaluated. In particular, compared to Mg-MS, a greater proportion of participants reported increased bowel movements with MgO and Mg-C, as well as a sense of gastric heaviness with Mg-BG. This indicated that the microencapsulation of MgO is a tool to reduce the main side effects associated with the intake of Mg-based supplements. Larger-scale studies will be considered to further evaluate the tested products in different populations, to conduct an in-depth analysis of the side effects, and to investigate broader applications and health-associated biomarkers.

## 5. Conclusions

Altogether, our results showed that Mg-MS, MgO, and Mg-C increased Mg levels in plasma upon oral intake. This clinical study introduced MAGSHAPE^TM^ microcapsules, an innovative microencapsulated form of Mg oxide, as a competitive alternative to existing Mg sources for food supplementation. Our findings demonstrated that Mg-MS improved the dynamics of Mg bioavailability after oral administration compared to other Mg sources, especially when compared to Mg bisglycinate, one of the gold standards for Mg supplementation. Moreover, the sustained release observed for Mg-MS highlights its suitability for applications where a prolonged increase in plasma Mg levels is required. Most importantly, the Mg-MS technology increases the levels of Mg in plasma to a greater extent than the non-encapsulated form of Mg oxide. This demonstrated that the microencapsulated Mg has a different absorption dynamic that maintains higher Mg levels in plasma up to, at least, 6 h. Finally, our results showed that the oral intake of MAGSHAPE^TM^ microcapsules is associated with fewer gastric and intestinal side effects compared to the other Mg sources, especially the non-encapsulated Mg oxide, thus highlighting the health-associated benefits of microencapsulation technology applied in this nutraceutical ingredient.

## Data Availability

The raw data supporting the conclusions of this article will be made available by the authors upon request.

## Supplementary Materials

The following supporting information can be downloaded at: https://www.mdpi.com/article/10.3390/nu16244367/s1. Figure S1: Graphical representation of the absolute Mg levels in plasma (mg L^−1^) in all volunteers in the assessed time points for each product; Figure S2: Graphical representation of the increase of absolute (in mg L^−1^) and normalized Mg levels in plasma after 1, 4 and 6 h of the oral intake compared to the basal levels, for each tested product; Figure S3: Bar graph representation of the increase of Mg levels in plasma in mg L^−1^ after 1, 4 and 6 h of the oral intake compared to the basal levels, for each tested product; Table S1: Restricted Mg-rich foods; Table S2: Volunteers’ information.
