# Supplementary material for: Comparative Clinical Study on Magnesium Absorption and Side Effects After Oral Intake of Microencapsulated Magnesium (MAGSHAPETM Microcapsules) Versus Other Magnesium Sources
Source: Nutrients. 2024 Dec 18;16(24):4367. doi: 10.3390/nu16244367 (PMC11677548; doi:10.3390/nu16244367)
Supplement: Supplementary file 1 [file nutrients-16-04367-s001.zip › nutrients-3325230-supplementary.pdf]

SUPPLEMENTARY MATERIALS

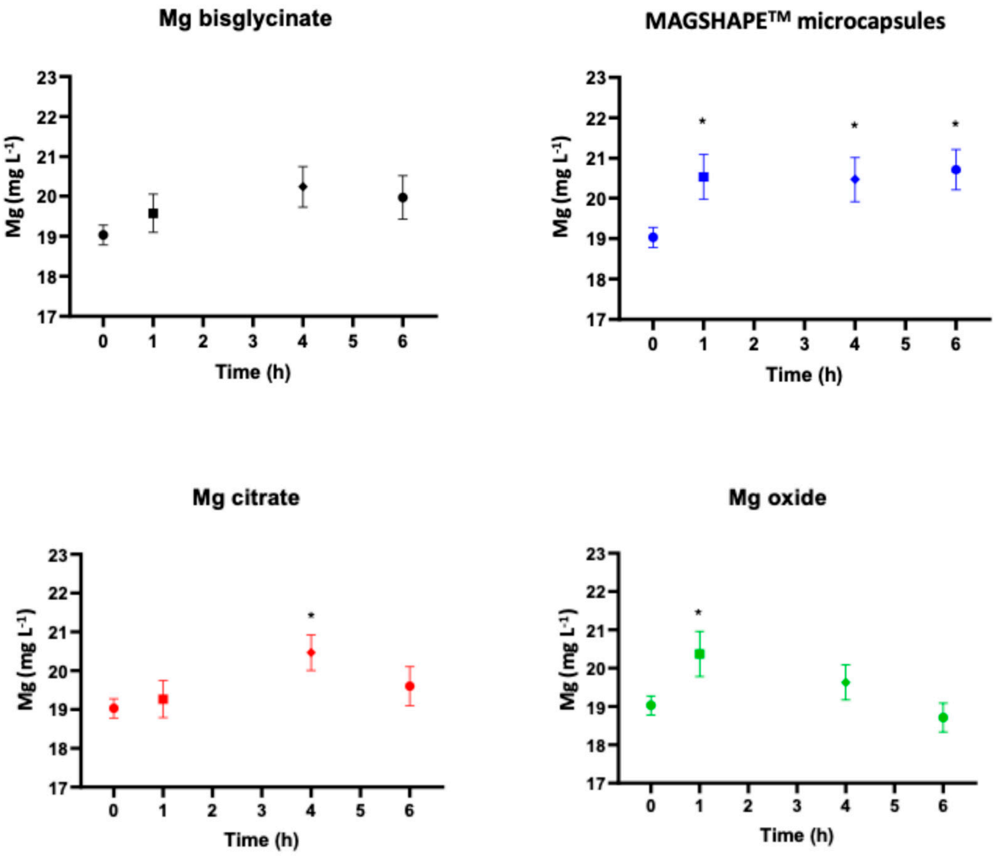

Figure S1. Graphical representation of the absolute Mg levels in plasma (mg L^{-1}) in all volunteers in the assessed time points for each product. Dara are represented as mean ± S.E.M. Asterisks indicate statistically significant differences compared to basal levels (0 h) as * p-value < 0.05.

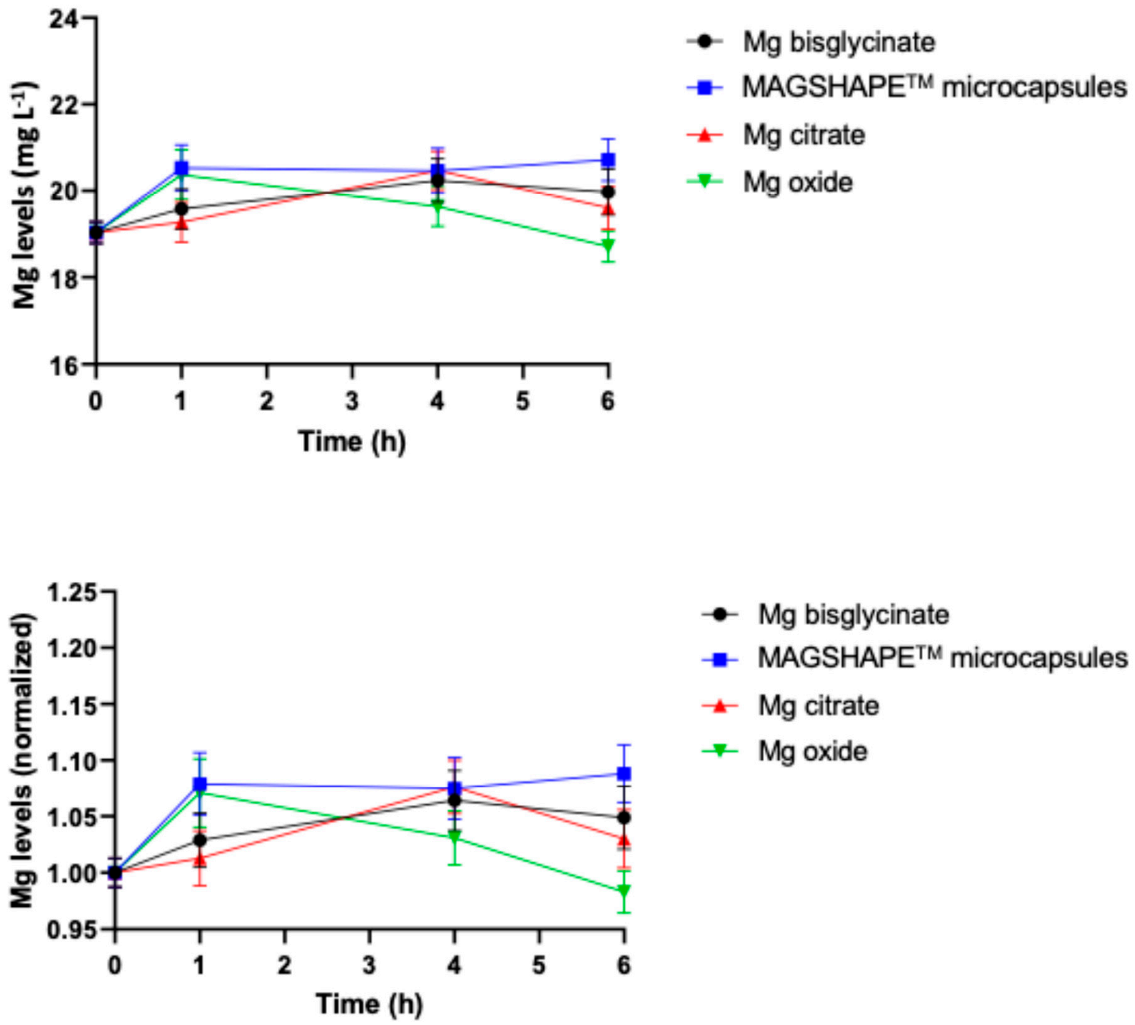

Figure S2. Graphical representation of the increase of absolute (in mg L^{-1}) and normalized Mg levels in plasma after 1, 4 and 6 h of the oral intake compared to the basal levels, for each tested product.

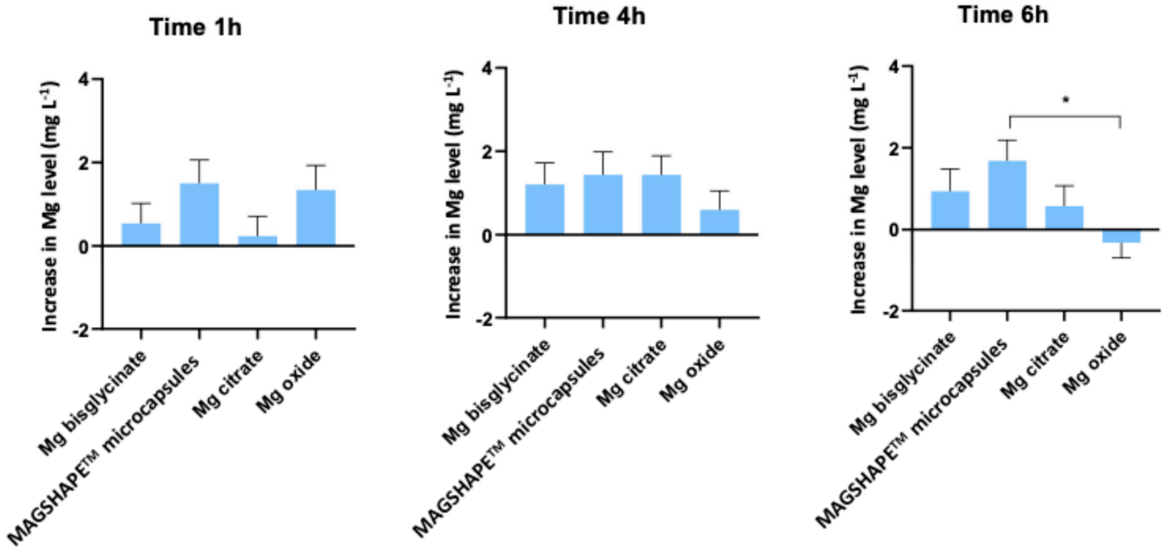

Figure S3. Bar graph representation of the increase of Mg levels in plasma in mg L^{-1} after 1, 4 and 6 h of the oral intake compared to the basal levels, for each tested product. Data are represented as mean ± S.E.M. Asterisks indicate statistically significant differences compared to basal levels (0 h) as * p-value < 0.05.

Table S1. Restricted Mg-rich foods.

**List of Mg-rich food excluded from the diet of the study subjects**
Whole grains, wheat bran, whole wheat bread
Wild rice and brown rice
Oats
Millet
Quinoa
Buckwheat
Cocoa and chocolate
Nuts: Almonds, hazelnuts, cashews, pine nuts, peanuts, macadamia, pistachios, nuts
Nougat, marzipan and pastries made with nuts
Dried legumes: beans, chickpeas, beans, beans, dried peas
Chickpea flour, soy flour.
Soy sauce
Flax seeds, sesame, poppy, hemp, chia
Sunflower seeds
Pumpkin seed
Squid, cuttlefish and the like
Caviar, fish roe, barnacles
Snails
Dry shitake
Skimmed milk powder
Prickly Pears
Dried figs
Chard
Spinach
Avocado
Dried basil
Dried coriander

Table S2. Volunteers' information.

Nº Volunteer	ID Volunteer	Age	Gender	IMC
1	2392	47	Female	20.0
2	2791	44	Female	22.5
3	2449	23	Female	21.5
4	3624	21	Male	27.4
5	3360	21	Female	24.8
6	1838	37	Female	24.4
7	3653	49	Female	26.4
8	3660	33	Male	32.7
9	2704	37	Female	23.3
10	3478	51	Male	27.6
11	3489	27	Female	20.0
13	3363	30	Female	23.4
14	3274	47	Female	24.6
15	2627	46	Female	23.4
16	2374	53	Female	26.4
17	2257	55	Female	21.8
18	2950	46	Female	24.5
19	2168	25	Female	25.2
20	3663	54	Female	29.1
21	2810	44	Female	26.0
23	2338	27	Female	31.4
24	2638	53	Male	30.4
26	3269	51	Female	28.1
27	3383	55	Male	28.3
28	3516	42	Female	21.5
29	3312	48	Male	25.6
30	3010	24	Male	20.0
31	3568	49	Male	26.4
32	2615	53	Female	26.6
33	2329	26	Female	23.1
34	2496	22	Male	28.1
35	2471	26	Male	34.7
36	90	53	Male	28.1
37	3221	37	Male	22.9
38	2356	28	Male	24.6
39	2650	25	Male	25.1
40	2886	25	Male	22.2
41	2472	23	Male	24.7
42	2938	49	Male	27.0
43	3655	22	Male	21.9
